# Charcot-Marie-Tooth neuropathy score and ambulation index are both predictors of orthotic need for patients with CMT

**DOI:** 10.1007/s10072-021-05646-9

**Published:** 2021-10-06

**Authors:** Valeria Prada, Riccardo Zuccarino, Cristina Schenone, Giulia Mennella, Marina Grandis, Michael E. Shy, Angelo Schenone

**Affiliations:** 1 Department of Neurosciences, Rehabilitation, Ophthalmology, Genetics and Maternal/Child Sciences, Genova, Italy; 2 Department of Neurology, Carver College of Medicine, University of Iowa, 200 Hawkins Drive, Iowa City, IA 52242-1009 USA; 3 Neuromuscular Omnicentre (NeMO) Trento-Fondazione Serena Onlus, Pergine Valsugana, TN Italy; 4 Ospedale Policlinico IRCCS San Martino, Genova, Italy

**Keywords:** Charcot-Marie-Tooth disease, CMT, Predictor, Ambulation, Braces, Orthotics

## Abstract

Charcot-Marie-Tooth (CMT) disease is the most common hereditary neuropathy with an estimated prevalence of 1 person affected on 2500. Frequent symptoms include distal weakness and muscle wasting, sensory loss, reduced deep tendon reflexes, and skeletal deformities, such as hammer toes and pes cavus*.* CMT is a progressive disease and patients’ needs change over their lifetime. In particular, ambulation aids are increasingly needed to maintain ambulation and reduce the risk of falls. We performed a retrospective analysis of medical records from 149 patients with confirmed CMT to evaluate patients ambulation needs related to the severity of their CMT as measured by the CMT Neuropathy Score (CMTNS) and Ambulation Index (AI). Most patients required some form of orthotics (86.6%). The CMTNS and AI scores both differed significantly between patients with no orthotics compared to those who wore insoles/inserts. The CMTNS and AI also differed significantly between patients wearing insoles and those with ankle foot orthotics (AFOs). CMTNS and the AI were valid predictors of the type and choice of the orthotics. Both the CMTNS and AI can be effective tools to aid in the correct choice of orthotics in patients affected by CMT.

**Supplementary Information:**

The online version contains supplementary material available at 10.1007/s10072-021-05646-9.

## Introduction

Charcot-Marie-Tooth (CMT) disease is the most common hereditary neuropathy with an estimated prevalence of 1 in 2500 [1, 2]. CMT is caused by mutations in more than 80 different genes [3–5]. Despite the genetic heterogeneity, most forms have common symptoms though the rates of progression may differ [6]. These symptoms include distal weakness and muscle wasting, distal loss of proprioception, sensory loss, reduced deep tendon reflexes, and skeletal deformities, such as hammer toes and pes cavus [7]. Taken together, these symptoms usually result in an abnormal gait, foot drop, and difficulties with balance in patients [8, 9]. Progression of these symptoms is variable ranging from slow [10] to more rapid depending on the specific genetic cause and mutation [5].

However, in all these cases, patients need to have their gait analyzed over a lifetime because the need and type of correction will change as disability increases [11]. Moreover, foot deformities and surgeries are variables that can influence the needs of orthotics over time [12–14].

Patients with CMT typically use different kinds of orthotics devices, ranging from shoe inserts to ankle foot orthoses (AFOs) [15]. We separate AFOs into two types. The first are the low AFOs that extend just above the ankle and provide stability only at the ankle, without using the tibia bone as a lever. The second type are the AFOs which extend higher up the calf and use the tibia as a lever (like leaf spring or toe off orthoses) [16, 17]. Currently, we are unaware of consensus or guidelines as when to use any of these devices. We hypothesize that having more complete information of the relationship between disease severity and the type of orthotic used would provide useful information for health care providers to help provide the best choice in what they prescribe for patients [16].

To begin addressing this issue, we performed a retrospective analysis of a cohort of 149 patients with CMT followed in the outpatient unit of the Neurological Clinic of the San Martino Hospital (Italy). The aim of the study was to identify correlations between the use of different insoles/orthoses and clinical outcome assessments (COA) of CMT so that we could develop preliminary algorithms that would be useful for managing the ambulation needs of our patients.

## Methods

A retrospective analysis was carried out on medical records maintained in the Inherited Neuropathy Facility of the Ospedale Policlinico San Martino IRCCS of Genova. Data was recorded anonymously and placed in a file excel, specifying the following: sex, date of birth, genetical diagnosis, date of the visit, type of orthosis, Ambulation Index (AI), and CMT Neuropathy Score version 2 (CMTNSv2) [18, 19]. We also recorded data for the AI, based on the time and needs of patients to walk 25 feet [20].

A total of 221 charts of confirmed diagnosis of CMT were examined. We excluded 72 patients because they lacked a CMTNSv2 or specifications about insoles or orthoses.

We therefore included in the study all the patients with (1) a specified and genetical diagnosis of CMT; (2) a CMTNSv2 score; (3) an AI score; and (4) a prescription of an insole or orthoses. All data came from the same visit.

### Statistical analyses

An ANOVA test with Tukey’s multiple comparisons test was made for comparison of different classes. A Spearman *r* test was used for correlation between scales. Analysis of the difference between two groups was carried out with a Mann–Whitney *t* test. Multiple comparisons were analyzed with the Tukey multiple comparisons test. Significance was considered for *p* < 0.05.

## Results

One hundred forty-nine patients (74 males and 75 females) were included in the study and met all the inclusion criteria. The mean age was 53.2 (± 16.7) with an age range between 18 and 89 years. Sixty-eight patients (45.6%) had CMT1A, 6 (4.0%) patients had CMT1B, 24 patients (16.1%) had CMT2 subtypes, and 3 (2.0%) had recessive CMT4 forms. Thirty patients (20.0%) were diagnosed with CMTX (29 CMTX1 and 1 CMTX5) and the remaining 18 patients (12.1%) had HNPP.

Most of the patients required some form of foot or ankle support; only 13.4% (*n* = 20) were not wearing any type of orthotic. Forty-eight percent (47.7; *n* = 71) wore just inserts or insoles. Low AFOs were used by 12.8% (*n* = 19), and higher AFOs by17.4% (*n* = 26). Seven patients (4.7%) also required a cane to walk and 6 patients (4.0%) needed a walker or a wheelchair for ambulation (Table 1). The mean and the range of the scores from the CMTNS and ambulation are shown in Table 2 for the different CMT subtypes. We identified strong correlations between the CMTNS and AI scales (*r* = 0.75; *r*^2^ = 0.49; *p* < 0.0001; Supplementary Fig. 1).

**Table 1:** Generalities of patients and distribution of CMT types

Generalities			
Patients (*N*)		149	
Mean age (years ± SD)		53.2 ± 16.7	
Age range		18–89	
M/F		74/75	
CMT types			
	% (*N*)	CMT subtypes	*N*
CMT1A	45.6 (68)		
CMT1B	4.0 (6)		
CMT2	16.1 (24)		
		CMT2A	5
		CMT2E	1
		CMT2F	4
		CMT2I	4
		CMT2J	10
CMT4	2.0 (3)		
		CMT4A	1
		CMT4D	1
		CMT4B2	1
CMTX	20.0 (30)		
		CMTX1	29
		CMTX5	1
HNPP	12.1 (18)		
Overall orthotics % (*N*)			
No orthotics	13.4 (20)		
Inserts/insoles	47.7 (71)		
Low AFO	12.8 (19)		
AFO	17.4 (26)		
One cane	4.7 (7)		
Walker/chair	4.0 (6)

**Table 2:** Ambulation Index and CMTNS of the different clusters of patients

	Mean age	Range age	Mean Ambulation Index	Range Ambulation Index	Mean CMTNS	Range CMTNS
Overall (149)	53.2 ± 16.7	18–89	1.5 ± 1.5	0–7	11.3 ± 6.7	1–28
CMT1A (68)	51.0 ± 17.4	18–89	1.3 ± 1.4	0–5	11.4 ± 5.8	1–27
CMT 1B (6)	49.3 ± 15.1	34–73	2.2 ± 2.5	0–7	16.2 ± 5.2	10–25
CMTX (30)	52.1 ± 16.5	24–84	1.9 ± 1.5	0–6	13.2 ± 6.6	3–25
CMT 2 (24)	66.3 ± 12.9	34–81	1.9 ± 1.2	0–4	11.4 ± 4.8	3–21
CMT 4 (3)	40.7 ± 17.6	21–55	4.7 ± 1.5	3–6	24.3 ± 3.5	21–28
HNPP (18)	49.7 ± 11.8	31–73	0.5 ± 1.0	0–3	4.3 ± 6.7	0–26

**Fig. 1:**
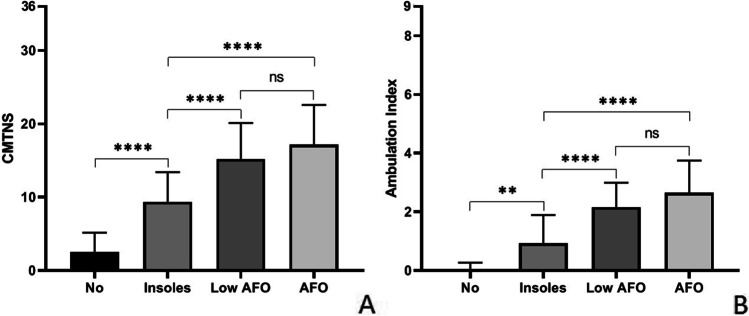
Orthotics compared with CMTNS and AI. **A** Mean and SD of the CMTNS in the different groups: no orthotics: 2.5 ± 2.6; insoles: 9.3 ± 4.1; low AFO: 15.2 ± 4.9; AFO: 17.1 ± 5.4. No orthotics group is statistically different from the insoles group (*p* < 0.0001); insoles group is statistically different from the low AFO group (*p* < 0.0001) and AFO group (*p* < 0.0001). No statistical differences have been found between the low AFO and AFO group (*p* = 0.45). **B** The mean and SD of the AI for every group is as follows: no orthotics worn 0.05 ± 0.2; insoles 0.93 ± 1.0; low AFO 2.2 ± 0.8; AFO 2.6 ± 1.1. There is a significant difference between the AI of people who do not wear orthotics and people who use insoles (*p* = 0.01), between people who wear insoles and people who wear low AFO (*p* < 0.0001), and between people who use insoles and people who wear AFO (*p* < 0.0001). No differences have been found in the group who wears the low AFO and the one who uses AFO (*p* = 0.27)

We next compared the type of orthoses used with the CMTNSv2 and found that CMTNS scores were significantly different between patients wearing no orthotics compared to those with insoles alone (patients without orthotics: 2.5 ± 2.6 CMTNS; patients with insoles: 9.3 ± 4.1 CMTNS; *p* < 0.0001). There were also significant differences between patients wearing insoles compared to those wearing low and high AFOs (patients with low AFO: 15.2 ± 4.9 CMTNS; high AFO: 17.1 ± 5.4; *p* < 0.0001 for both). Although there were slight differences between the group wearing low and high AFOs, these differences did not reach significance (*p* = 0.45; Fig. 1A).

Similar results were obtained with correlations to the AI (Fig. 1B). The mean AI for the three groups was as follows: no orthotics worn 0.05 ± 0.2; insoles only 0.9 ± 1.0; low AFO 2.2 ± 0.8; high AFO 2.6 ± 1.1. The difference between the AI in those who wore not orthotics and those who wore insoles was significant (*p* = 0.01), as were the differences between individuals who wore insoles compared to those who wore low AFOs (*p* < 0.0001) and high AFOs (*p* < 0.0001). No significant differences were found between the group who wore low AFOs compared to the group with high AFOs (*p* = 0.27; Fig. 1B).

**Fig. 2:**
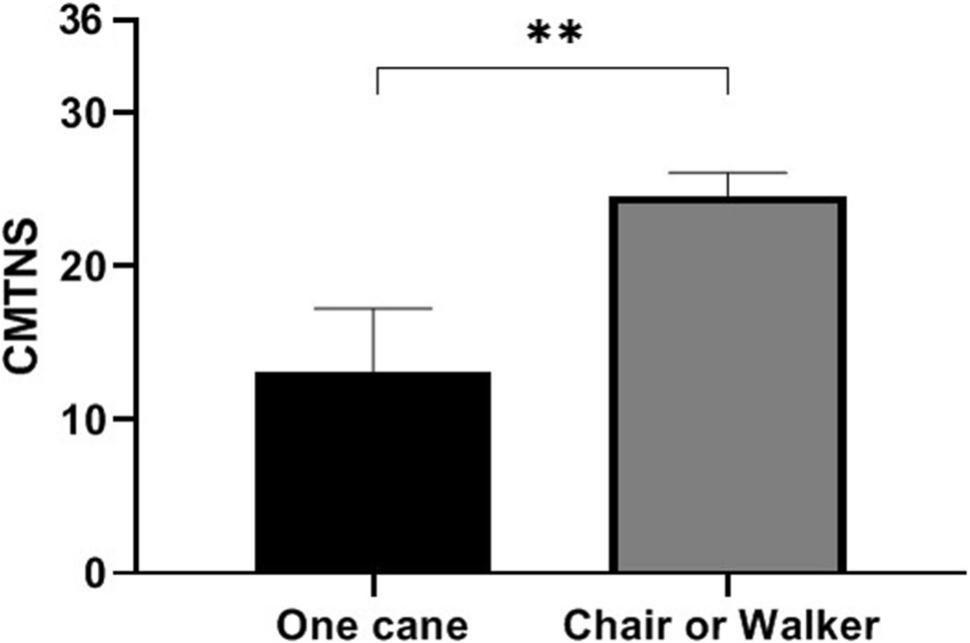
CMTNS in patients with severe problems in walking. Patients who need one cane: 13.1 ± 4.1 CMTNS score. Patients who need a wheelchair or a walker: 24.6 ± 1.5 CMTNS score (*p* = 0.001)

We then evaluated patients who were using canes, walkers, or wheelchairs, though these numbers were small. Patients who needed a cane for walking had significantly lower CMTNS compared to those who needed a walker or a wheelchair (one cane: 13.1 ± 4.1; chair or walker 24.6 ± 1.5; *p* = 0.001; Fig. 2).

**Fig. 3:**
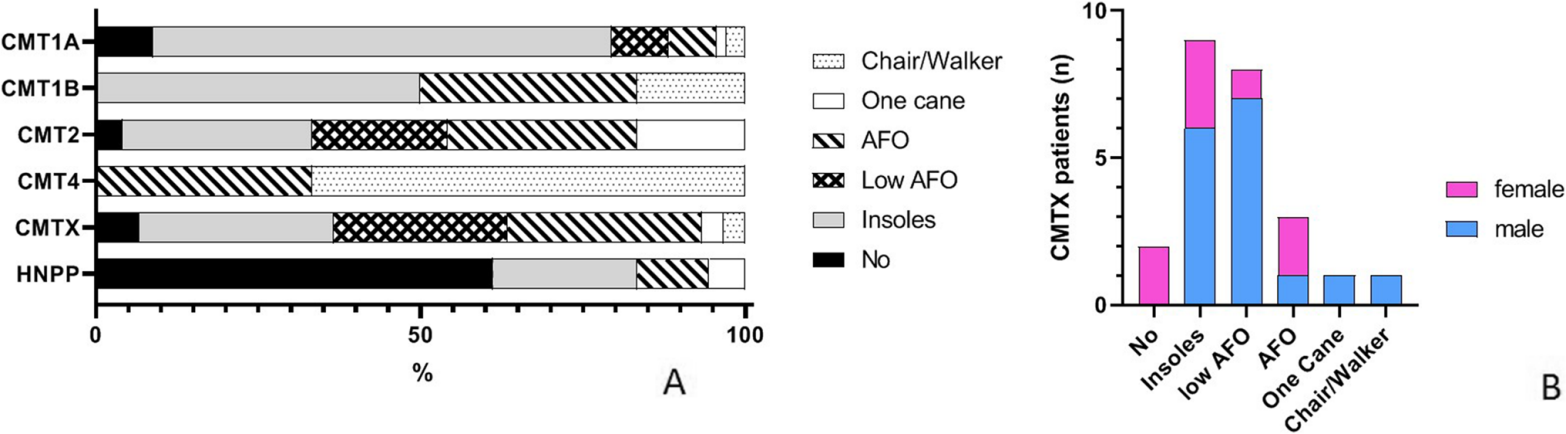
Distribution of orthotics in the different types of CMT. **A** In CMT1A, 8.8% (*n* = 6) does not wear any kind of orthotics; 70.6% (*n* = 48) wears insoles/inserts; 8.8% (*n* = 6) wears low AFO and 7.3% (*n* = 5) wears AFO; 1.5% (*n* = 1) uses one cane to walk; and 2.9% (*n* = 2) needs a wheelchair or a walker. In CMT1B patients, 50.0% (*n* = 3) wear insoles/inserts; 33.3% (*n* = 2) wear AFO; and 33.3% (*n* = 1) need a wheelchair or a walker. In CMT2, 4.2% (*n* = 1) does not wear any kind of orthotics; 29.2% (*n* = 7) wears insoles/inserts; 20.8% (*n* = 5) wears low AFO and 29.2% (*n* = 7) wears AFO; and 16.7% (*n* = 4) uses one cane to walk. In CMT4, 33.3% (*n* = 1) wears AFO and 66.7% (*n* = 2) needs a wheelchair or a walker. In CMTX, 6.7% (*n* = 2) does not wear any kind of orthotics; 30.0% (*n* = 9) wears insoles/inserts; 26.7% (*n* = 8) wears low AFO and 30.0% (*n* = 9) wears AFO; 3.3% (*n* = 1) uses one cane to walk; and 3.3% (*n* = 1) needs a wheelchair or a walker. In HNPP, 61.1% (*n* = 11) does not wear any kind of orthotics; 22.2% (*n* = 4) wears insoles/inserts; 11.1% (*n* = 2) wears AFO; and 5.6% (*n* = 1) uses one cane to walk. **B** Distribution between males and females in CMTX. Two females do not need any orthotics, inserts are worn by 6 males and 3 females, low AFOs are needed by 7 males and 1 female, and normal AFOs are used by 7 males and 2 females. One male needs to walk with the aid of one cane and 1 male needs a chair/walker

Finally, we studied the distribution of the different orthoses between the different CMT subtypes. In CMT1A and CMT1B, the most frequently used orthotics were insoles (CMT1A 70.6%, *n* = 48; CMT1B 50.0%, *n* = 3). Patients with CMT2 had a different distribution: 29.2% (*n* = 7) wore insoles, 20.8% (*n* = 5) wore low AFO, 29.2% (*n* = 7) wore high AFOs, 16.7% (*n* = 4) used one cane, and only 4.2% (*n* = 1) did not use orthoses. The majority of our CMT4 patients used wheelchairs or walkers (66.7%, *n* = 2), and only 1 patient wore an AFO (33.33%) and used insoles. 11.1% (*n* = 2) needed AFOs and 1 patient (5.6%) walked with one cane (Supplementary Table 1; Fig. 3A).

We paid particular attention to the patients with CMTX (29 CMTX1 and 1 CMTX5) because we had a large group of these patients and because males are often more severely affected than females. Two patients, both females, did not wear any orthotic (6.7%). Nine patients with CMTX (30.0%) used inserts (6 males, 3 females), low AFO were worn by 8 (26.7%) patients (7 males, 1 female), and high AFOs were used by 9 patients (30.0%, 7 males, 2 females). One with CMTX (3.3%) used a cane to walk and an additional patient used a wheelchair. Both of these patients were male (Fig. 3B).

## Discussion

We have retrospectively evaluated orthotic use in 149 patient subjects with different confirmed types of CMT. Consistent with the literature, our largest subgroup had CMT1A [21, 22]. Both genders were equally represented. Most of our patients (86.6%) required orthotics of some type highlighting the importance of inserts or AFOs in managing CMT. This is in keeping with our concept that the use of orthotics and orthoses is important for CMT patients because it facilitates a better quality of gait and balance [23, 24]. In addition, the choice of an appropriate orthotic is important in progressive disorders like CMT in which ambulation needs change with disease impairment, as well as with alterations in foot structure and foot and ankle surgeries. A poor choice in the type of orthotics/orthoses used will likely result in exacerbating difficulties in ambulation [14], decreased likelihood that patients will wear the orthotic, and increased risk in tripping and falling during daily activities [8].

The literature suggests that most patients are satisfied overall with the orthotics they are wearing[17]. However, even in this group there were often concerns from patients about how their orthotics could be improved [17], and this is important because these devices are fundamental for many activities of daily living [25]. Specifically, we found that correlations with the CMTNS and AI accurately both predicted the type of orthotic worn by patients, consistent with prior studies that the CMTNS and AI correlate with each other [18]. The correlations with the CMTNS and AI are also consistent with what was predicted in the original CMTNS report in which neuropathies were characterized as mild (CMTNS ≤ 10), moderate (CMTNS 11–20), and severe (≥ 21) [18]. We found in our study people wearing no orthotics typically fell into the mild neuropathy scoring. Among people who used orthotics, 47.7% used only insoles and 30.2% utilized low or high AFOs. Those who had CMTNS between 10 and 15 usually wore only insoles, those with scores up to 20 most often wore low AFOs, and those with scores above 20 either wore high AFOs, like the leaf spring, or rarely needed walkers or wheelchairs. We recognize that the “motor symptoms legs” component of the CMTNSv2 includes orthotics as part of the scoring [19]. However, this represents just one of the nine items in the CMTNS so we believe this one item cannot be responsible for all the correlation with orthotic use and the CMTNSv2. The AI also predicted well the type of orthotic worn by patients as correlations with these scores and the different orthotics were also significant, even, in this case, the results could strongly depend on the fact that, in this scale, orthotics are one on the criteria to consider in the scoring.

We were not able to predict the type of orthotic used based on CMT subtype, probably because there was variability in severity of neuropathy within the different subtypes including CMT1A, CMT1B, CMTX, and CMT2. The one potential exception was in patients with recessive forms of CMT such as CMT4, who were usually more severely affected and were likely to use high AFOs or even walkers and wheelchairs.

We did pay special attention to differences between men and women with CMTX (mostly of them affected by CMTX1), since women are often more mildly affected than men due to the presence of a normal X-chromosome in addition to the mutated allele [26]. In agreement, men with CMTX were more likely to use high AFOs or even a cane/walker compared to women.

In conclusion, we find that the choice of the correct AFO for patients really depends on the severity of their neuropathy, which can be predicted by their CMTNS or AI. We recognize potential contributions to orthotic needs by anatomical foot structure and/or previous surgical interventions. However, our data demonstrate that the CMTNS and AI are both good predictors of orthotic needs for patients with CMT.

## Supplementary Information

Below is the link to the electronic supplementary material.

Supplementary file1 (JPG 27 kb)

Supplementary file2 (DOCX 15 kb)

## Data Availability

The datasets generated and analyzed during the current study are not publicized but are available from the corresponding author on reasonable request.
