# Supplementary figures and images for: Charcot-Marie-Tooth neuropathy score and ambulation index are both predictors of orthotic need for patients with CMT
Source: Neurol Sci. 2021 Oct 6;43(4):2759–64. doi: 10.1007/s10072-021-05646-9 (PMC8918134; doi:10.1007/s10072-021-05646-9)

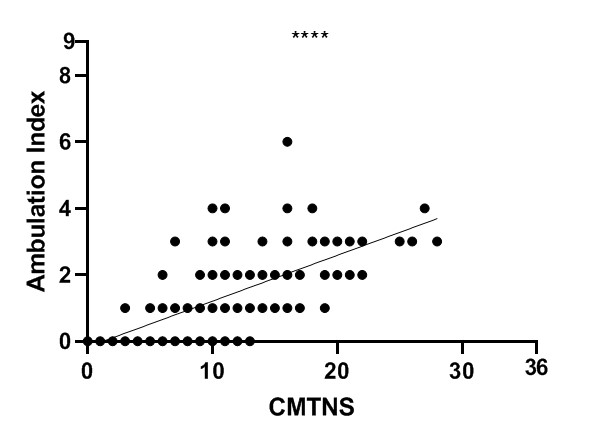

Supplement: Supplementary file 1 — Supplementary file1 (JPG 27 kb) [file 10072_2021_5646_MOESM1_ESM.jpg]
