# Supplementary material for: Charcot-Marie-Tooth neuropathy score and ambulation index are both predictors of orthotic need for patients with CMT
Source: Neurol Sci. 2021 Oct 6;43(4):2759–64. doi: 10.1007/s10072-021-05646-9 (PMC8918134; doi:10.1007/s10072-021-05646-9)
Supplement: Supplementary file 2 — Supplementary file2 (DOCX 15 kb) [file 10072_2021_5646_MOESM2_ESM.docx]

Supplementary Material

Supplementary Table 1 – Percentage of orthotics in the different forms of CMT.

|  | No orthoses | Insoles | Low AFO | AFO | One cane | Chair or walker |
| --- | --- | --- | --- | --- | --- | --- |
| CMT1A | 8.8 | 70.6 | 8.8 | 7.4 | 1.5 | 2.9 |
| CMT1B | 0.0 | 50.0 | 0.0 | 33.3 | 0.0 | 16.7 |
| CMT2 | 4.2 | 29.2 | 20.8 | 29.2 | 16.7 | 0.0 |
| CMT4 | 0.0 | 0.0 | 0.0 | 33.3 | 0.0 | 66.7 |
| CMT1X | 6.7 | 30.0 | 26.7 | 30.00 | 3.3 | 3.3 |
| HNPP | 61.1 | 22.2 | 0.0 | 11.1 | 5.6 | 0.0 |

Supplementary Legend

Supplementary Figure 1. Figure 1. Correlation between AI and CMTNS scores. There is a significant correlation between AI and CMTNS scores (r= 0.75; r^2^=0.49; p<0.0001).
